# Maternal weight, gut microbiota, and the association with early childhood behavior: the PREOBE follow-up study

**DOI:** 10.1186/s13034-023-00589-9

**Published:** 2023-03-21

**Authors:** Ana Nieto-Ruiz, Tomás Cerdó, Belén Jordano, Francisco J. Torres-Espínola, Mireia Escudero-Marín, María García-Ricobaraza, Mercedes G. Bermúdez, José A. García-Santos, Antonio Suárez, Cristina Campoy

**Affiliations:** 1 Department of Paediatrics, Faculty of Medicine, University of Granada, Avda. Investigación 11, 18016 Granada, Spain; 2 Biomedical Research Centre, EURISTIKOS Excellence Centre for Paediatric Research, University of Granada, 18016 Granada, Spain; 3 Instituto de Investigación Biosanitaria de Granada (Ibs.GRANADA), San Cecilio University Hospital. Health Sciences Technological Park, 18016 Granada, Spain; 4 Maimonides Biomedical Research Institute of Córdoba (IMIBIC), Reina Sofia University Hospital, University of Córdoba, Córdoba, Spain; 5 Clinical University Hospital San Cecilio. Paediatric Service, Granada, Spain; 6 Neurosciences Institute Dr. Federico Oloriz - University of Granada. Health Sciences Technological Park, Avda. del Conocimiento, S/N., 18016 Granada, Spain; 7 Department of Biochemistry and Molecular Biology 2, School of Pharmacy, University of Granada, Granada, Spain; 8 Institute of Nutrition and Food Technology (INYTA), Biomedical Research Centre, University of Granada, Health Sciences Technological Park, Avda. del Conocimiento, S/N., 18016 Granada, Spain; 9 Spanish Network of Biomedical Research in Epidemiology and Public Health (CIBERESP), Granada’s Node, Institute of Health Carlos III, 28029 Madrid, Spain

**Keywords:** Obesity, Gestational diabetes mellitus, Pregnancy, Microbiota-gut-brain axis, Early programming, Children, Behavioral problems, CBCL, Breastfeeding

## Abstract

**Background and aim:**

Maternal overweight and breastfeeding seem to have a significant impact on the gut microbiota colonization process, which co-occurs simultaneously with brain development and the establishment of the “microbiota-gut-brain axis”, which potentially may affect behavior later in life. This study aimed to examine the influence of maternal overweight, obesity and/or gestational diabetes on the offspring behavior at 3.5 years of age and its association with the gut microbiota already established at 18 months of life.

**Methods:**

156 children born to overweight (OV, n = 45), obese (OB, n = 40) and normoweight (NW, n = 71) pregnant women participating in the PREOBE study were included in the current analysis. Stool samples were collected at 18 months of life and gut microbiome was obtained by 16S rRNA gene sequencing. Behavioral problems were evaluated at 3.5 years by using the Child Behavior Checklist (CBCL). ANOVA, Chi-Square Test, ANCOVA, Spearman’s correlation, logistic regression model and generalized linear model (GLM) were performed.

**Results:**

At 3.5 years of age, Children born to OV/OB mothers showed higher scores in behavioral problems than those born to NW mothers. Additionally, offspring born to OB mothers who developed gestational diabetes mellitus (GDM) presented higher scores in *attention/deficit hyperactivity* and *externalizing problems* than those born to GDM OV/NW mothers. *Fusicatenibacter* abundance found at 18 months of age was associated to lower scores in *total, internalizing and pervasive developmental problems*, while an unidentified genus within *Clostridiales* and *Flavonifractor* families abundance showed a positive correlation with *anxiety/depression* and *somatic complaints*, respectively. On the other hand, children born to mothers with higher BMI who were breastfed presented elevated *anxiety*, *internalizing problems*, *externalizing problems* and *total problems* scores; likewise, their gut microbiota composition at 18 months of age showed positive correlation with behavioral problems at 3.5 years: *Actinobacteria* abundance and *somatic complaints* and between *Fusobacteria* abundance and *withdrawn behavior* and *pervasive developmental problems*.

**Conclusions:**

Our findings suggests that OV/OB and/or GDM during pregnancy is associated with higher behavioral problems scores in children at 3.5 years old. Additionally, associations between early life gut microbiota composition and later mental health in children was also found.

**Supplementary Information:**

The online version contains supplementary material available at 10.1186/s13034-023-00589-9.

## Introduction

Women of childbearing age have not been spared in the obesity epidemic worldwide. In fact, maternal obesity or overweight are among the most common conditions present during pregnancy in the developed world, affecting up to 47% of women [1]. Obesity in pregnancy has been associated with an increased risk of serious adverse outcomes, including miscarriage, fetal congenital anomaly, thromboembolism, gestational diabetes mellitus (GDM), preeclampsia, postpartum hemorrhage (PPH), wound infections, stillbirth and neonatal death and a lower breastfeeding rate [1–3]. The consequences of fetal exposure to the intrauterine conditions associated with maternal obesity may also extend to the neonate, as infants born to obese women appear to be at increased risk for a number of congenital anomalies, including neural tube defects and macrosomia [4, 5]. Additionally, it is important to note the effect of “nutrition programming” [6]. In fact, there is increasing evidence about the influence of maternal nutritional status and diet during pregnancy and infant type of feeding during the first months of life on child development and health in later adulthood [7]. Early programmed effects on anthropometric, metabolic, and neurological development have been suggested [8–10]. In this regard, milk from obese or gestational diabetic mothers presented changes in the concentration of several bioactive components [11] that could influence short- and long-term infant health status [12]. Furthermore, several studies have linked maternal and child weight through the transmission of maternal commensal microbiota, which is probably colonized during gestation [13–15]. Interestingly, Guilley et al. examined maternal human milk oligosaccharides (HMOs) between overweight/obese and normoweight mothers and observed an alteration in their composition. Furthermore, offspring from obese mothers presented a lower abundance of short-chain fatty acid (SCFA)-producing bacteria and lower fecal butyric acid levels, with a prediction of elevated adiposity at 12 months [16]. It follows that maternal obesity might also “program” offspring for lifelong obesity and associated metabolic and mental disorders [9, 17–19].

On the other hand, GDM is estimated to affect up to 14% of pregnancies [20] and is increasing worldwide in the wake of the increase in obesity [21]. Maternal consequences include an increased rate of hypertensive disorders during pregnancy and a future risk of type 2 diabetes mellitus (T2DM) as well as other aspects of metabolic syndrome, such as obesity, cardiovascular morbidities, recurrent GDM [1, 22] and a variation in the gut microbiota composition of the offspring during the first week of life and 9 months after birth [23]. Moreover, there are maternal complications secondary to delivering a neonate that is macrosomic or large for gestational age (LGA), such as an increased rate of cesarean delivery, postpartum hemorrhage and birth trauma [22].

The WHO [24] estimates that one in five children and adolescents experience mental health problems, which are known to predict other negative outcomes in later life, including noncompletion of schooling, physical health problems, drug and alcohol misuse, marital difficulties, increased mortality, injury risk, and involvement in the criminal justice system [25–28]. According to several authors, many risk factors during the prenatal period (a critical window for later behavioral development) could contribute to the development of future mental diseases [29–31]. Research also demonstrates the importance of environmental influences (e.g., prenatal and perinatal risk factors) on the causation of externalizing behavior [32]. One reason that may cause an increase in these risk factors is the inflammatory milieu present in maternal obesity and GDM during gestation [33, 34]. It has been proven that a fetus maturing under those conditions (obesity and/or GDM) has a higher risk of developing different neurodevelopmental disorders during childhood [35], such as attention problems, hyperactivity and anxiety [36–39]. Furthermore, adverse birth outcomes, including low birth weight and preterm birth, exposure during pregnancy to maternal smoking, alcohol consumption, GDM and psychological stress, have been identified as the most important pre- and perinatal factors associated with attention-deficit/hyperactivity disorder (ADHD) [40–42]. In a recent review, the association between maternal obesity and neurodevelopmental and psychiatric morbidity in offspring was investigated [43]. Brion et al*.* found significant associations between maternal overweight and externalizing and total problems at 3 years old in the Dutch Generation R cohort but not in the Avon Longitudinal Study [44]. Furthermore, Rodríguez et al*.* reported that a higher maternal prepregnancy body mass index (BMI) (overweight and obesity) was associated with core symptoms of ADHD in school-age children [45]. On the other hand, one hypothesis to explain the influence of maternal weight on their children is the transmission of obesogenic microbes from the mother to her offspring [13]. In this connection, several studies have shown that maternal prepregnancy obesity imprints a selective gut microbial composition during late infancy [46, 47].

During the first 3 years of life, children’s brains growing rapidly parallel to deep gut microbiota establishment and development through communication along the “gut-brain axis” has been postulated as one plausible mechanism influencing infant neurodevelopment [48, 49]. Experiments in animal models have shown that the maturation process of the gut microbiota coincides with intense synaptogenesis and pruning in the cerebral cortex, ending in adolescence [50–52]. Therefore, it is increasingly considered that gut microbes are part of the unconscious system influencing early neurodevelopment with potential later psychiatric expressions. In this regard, the theory that health across the lifespan is shaped during early sensitive windows, known as the developmental origins of health and disease, is a focus of pediatric molecular epidemiology, and the microbiome likely plays a crucial role in the most significant problems of behavior, such as externalizing (aggression, conduct problems, hyperactivity and inattention) and internalizing problems (emotional and affective problems, anxiety and depression) at 3 to 5 years old. Studying early biomarkers and symptomatology is of crucial importance because it could help to understand the underlying etiology of behavioral problems in preschool children [53, 54]. The objective of this study was to evaluate the influence of preconceptional maternal BMI and/or GDM on child behavior at 3.5 years old and to identify other possible influencing factors, including early gut microbiota composition and functionality and breastfeeding.

## Material and methods

### Study design and subjects

**Fig. 1:**
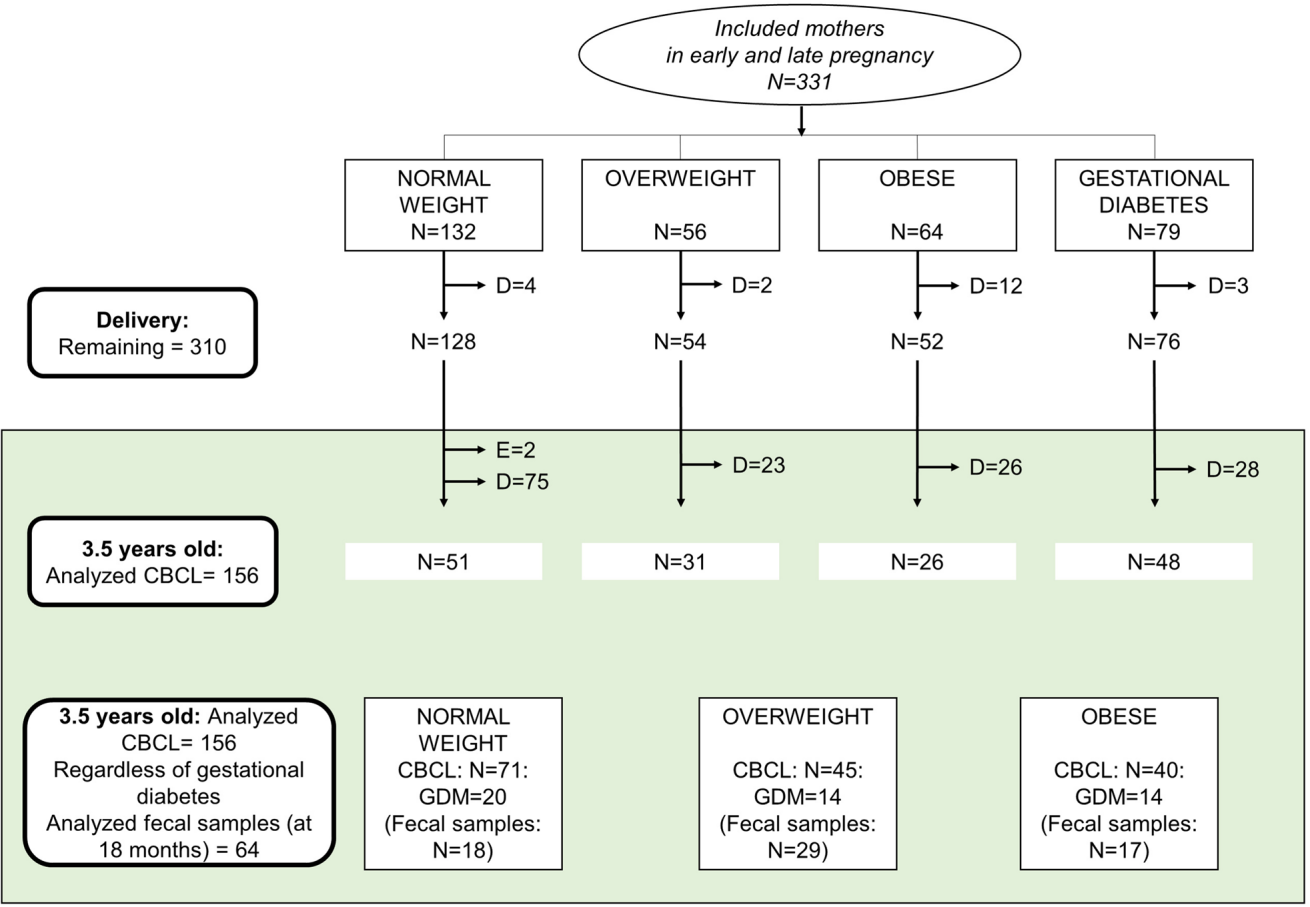
This figure shows drop-outs since delivery to 3.5 year old. D = drop-outs and E = exclusions (1 infant at delivery due to congenital heart disease and one before the six months follow up due to severe immunodeficiency). CBCL = Child Behavior Checklist; GDM = Gestational diabetes mellitus

The PREOBE study design, the characteristics of the pregnant women and their compliance have been described previously [18]. Briefly, 331 pregnant women were recruited between 2008 and 2012 through collaboration with the Clinical University Hospital San Cecilio and the Mother–Infant University Hospital of Granada, Spain, and their peripheral health centers. The inclusion criteria were single pregnancy at 12 to 34 weeks of gestation (before 20 weeks), aged 18 to 45 years, no simultaneous participation in another study, no drug treatment, no vegan diet, and no diagnosed diseases other than obesity, overweight or GDM. Based on their prepregnancy BMI, pregnant women were assigned to one of the following three groups: 1. normoweight (18.5 ≤ BMI < 25), n = 71; 2. overweight (25 ≤ BMI < 30), n = 45; and 3. obese (BMI ≥ 30, n = 40) (Fig. 1). Women who developed GDM during pregnancy remained in their group depending on prepregnancy BMI as follows: 1. normoweight group with GDM: n = 20; 2. overweight with GDM: n = 14; and 3. obese with GDM: n = 14 (Fig. 1). According to the hospital routines, mothers diagnosed with GDM were invited to participate in an endocrine nutritional program to optimize glucose control using nutritional and lifestyle recommendations and, in some cases, medical treatment. Overweight and obese mothers without GDM received no intervention or dietary recommendations except the regular ones.

### Ethics, consent, and permissions

Ethical approval was obtained from the Research Ethics Committee of the University of Granada. The Bioethical Committees for Clinical Research of the Clinical University Hospital San Cecilio and the Mother–Infant University Hospital of Granada, Spain, approved the project. A member of the research group provided full information about the project, and written informed consent was obtained from all women prior to entering the study. The project was registered at www.ClinicalTrial.gov (no.: NCT01634464).

### Data collection during pregnancy

In this study, baseline and background characteristics of pregnant women and their offspring, including maternal age, weight gain during pregnancy, preconceptional maternal weight, height, and BMI, maternal education level, maternal intelligence quotient (IQ) assessed with Catell’s Culture Fair Intelligence Test (g factor) [55, 56], smoking habit and alcohol consumption during pregnancy, gestational age at delivery, neonatal anthropometric measures (birth weight, length and head circumference), Apgar scores, family situation, type of delivery, baby gender, and parity, were collected using questionnaires and medical records. In addition, at three months of age, mothers were interviewed by an expert pediatrician about infant diet, which was categorized as breastfeeding, formula feeding or mixed feeding.

### Child behavior checklist (CBCL)

Parents completed the Child Behavior Checklist for Ages 1½–5 (CBCL) (Spanish validated version) [57] when their children were 3.5 years old. This test includes 101 items divided into two scales (“internalizing problems” and “externalizing problems”) and a total score. The internalizing problems scale is divided into four syndrome subscales: emotionally reactive behavior, anxiety/depression, somatic complaints, and withdrawn behavior. The externalizing problems scale is divided into two syndrome subscales: attention problems and aggressive behavior. Additionally, it assesses sleep, affective problems, anxiety, pervasive developmental problems, ADHD and oppositional defiant problems. The sum of the items of each CBCL scale provides a score that becomes the basis for the assignation of the children into one of the following three groups: normal, borderline and clinical (pathological) [57].

### DNA Extraction from stool samples

Genomic deoxyribonucleic acid (DNA) was extracted from the fecal bacteria of these infants (n = 64) at 18 months of age, as previously described [58]. Briefly, fecal samples were resuspended in 1 mL of TN150 buffer (10 mM Tris–HCl with a pH of 8.0 and 150 mM NaCl). Zirconium glass beads (0.3 g) and 150 mL of buffered phenol were added, and bacteria were disrupted with a mini bead beater set to 5000 rpm at 4 °C for 15 s (Biospec Products, Bartlesville, OK, USA). After centrifugation, genomic DNA was purified from the supernatant using a phenol‒chloroform extraction. Quality was checked by agarose gel electrophoresis and quantified with a QuantiT PicoGreen dsDNA assay kit (Invitrogen, Darmstadt, Germany).

### 16S rRNA gene sequencing and data processing

Genomic DNA from fecal bacteria was used as a template for 16S ribonucleic acid ribosomal (rRNA) gene amplification using 27F and 338R universal primers and two consecutive polymerase chain reactions (PCRs) to integrate Illumina multiplexing sequences as previously described [59]. The library was prepared by pooling equimolar ratios of amplicons and was sequenced using an Illumina MiSeq platform (Genetic Service, University of Granada, Granada, Spain). Reads were demultiplexed and sorted, and paired ends were matched to yield 240 nt reads. The dataset was filtered, and OTUs were defined at 99% similarity with the MOTHUR programs unique.seqs and pre.cluster [60]. Taxonomic classifications of OTUs were assigned using the naïve Bayesian algorithm CLASSIFIER of the Ribosomal Database Project [61]. OTUs were considered unassigned when the confidence value score was lower than 0.8 and were annotated using upper taxonomic ranks.

### Statistical analysis

All statistical analyses were performed using the SPSS statistical software package for Windows (version 24.0; IBM SPSS Inc., Chicago, IL, USA). Normally distributed variables are presented as the mean and standard deviation (SD), and nonnormal variables are presented as the median and interquartile range (IQR). Categorical variables are shown as frequencies and percentages. Differences in CBCL scores among the three PREOBE groups were evaluated using an analysis of variance (ANOVA) or Kruskal‒Wallis rank-sum test for nonnormal continuous variables and a chi-square or Fisher’s test for categorical variables. To determine differences according to the development of GDM, a univariate analysis of variance was performed. When significant differences were found between groups, a posteriori Bonferroni correction was used to identify significant pairwise group differences. A logistic regression model (enter method) was used to calculate odds ratios (ORs) and 95% confidence intervals (CIs) of having a normal/clinical pathologic value in the different groups of study using the normoweight group as a reference. Moreover, variables showing significant group differences were adjusted to univariate general linear models (ANCOVA) and multivariate logistic regression models. Spearman’s correlation analyses were performed to estimate the associations between preconceptional maternal BMI, breastfeeding, and child behavioral problems. In a secondary analysis, to establish the influence of confounder variables regardless of group, we used a logistic regression (Wald test). For the appropriate analysis of gut microbiota compositional data, we performed all statistical analyses and graphical representations in R [62] using the packages tidyverse [63], corrplot [64], psych and stats [65]. The contribution of metadata variables to microbiota community variation was determined by distance-based redundancy analysis (dbRDA) on genus-level Aitchison distance (Euclidian distance between samples after centered log-ratio transformation, as recommended for compositional data) with the capscale function in the vegan R package [64]. Correlations between gut microbial composition and CBCL scale were identified using Spearman’s nonparametric correlation test. The Bonferroni procedure (FDR < 0.01) was used to correct for multiple testing in correlations between taxa and CBCL scores. Associations between taxa and CBCL scores after screening the effect of microbiota covariates were assessed by fitting a generalized linear model (GLM) with the glm R function. Unless otherwise stated, the significance cutoff was set at p < 0.05 or false discovery rate (FDR) < 0.05 when multiple test correction was applied.

## Results

### Characteristics of the PREOBE study participants at 3.5 years old

Of the 331 pregnant women included in the PREOBE study, 156 were included in the present study after exclusions and dropouts. Children’s behavior was assessed at 3.5 years old using the CBCL test (Fig. 1).

Background and baseline characteristics of the mothers and their offspring are shown and compared among the three PREOBE groups in Table 1. Statistical analysis revealed significant differences between study groups in weight gain during pregnancy, maternal educational level and maternal IQ. In fact, normoweight pregnant women presented more weight gain during pregnancy than obese pregnant women (p = 0.008). Moreover, normoweight pregnant women showed a higher cultural level than obese pregnant women (p = 0.001) and higher IQ than overweight pregnant women (p = 0.025). On the other hand, obese mothers had an increased risk of GDM (35%) compared with overweight (31.11%) or normoweight mothers (28.17%), although the difference was not significant. Finally, differences in neonatal birth weight were marginally significant among groups (p = 0.065), as was the type of feeding at 3 months (p = 0.060). The skewed variables were included in the analyses.

**Table 1:** General characteristics of the PREOBE mother–child pairs who participated in the children behavioral follow up at 3.5 years of age

		**Normal weight (n = 71)**	**Overweight (n = 45)**	**Obese** **(n = 40)**	**P**^**1**^
Maternal age (y)^2^		31.93 ± 3.90	32.69 ± 4.56	31.55 ± 5.43	0.489
Pre-conceptional BMI (kg/m^2^)^2^		22.09 ± 1.72^a^	27.31 ± 1.32^b^	34.47 ± 4.19^c^	** < 0.001**
GWG (kg)^2^		11.32 ± 6.50^a^	9.95 ± 6.07^a,b^	6.64 ± 7.58^b^	**0.008**
GDM^3^	Yes	28.17%	31.11%	35.00%	0.754
Parity^3^	0	55.71%	52.27%	40.00%	0.275
	> 1	44.29%	47.73%	60.00%	
Family situation^3^	Single/Separate	1.41%	2.27%	5.00%	0.290
	Lives in partnership/ Married	98.59%	97.73%	92.50%	
	Others	0.00%	0.00%	2.50%	
Maternal Educational level^3^	Primary/Secondary	39.44%^a^	61.36%^a,b^	75.00%^b^	**0.001**
	University	60.56%^a^	38.64%^a,b^	25.00%^b^	
Maternal IQ (points)^2^		108.87 ± 12.16^a^	102.16 ± 13.73^b^	106.11 ± 12.51^a,b^	**0.025**
Paternal IQ (points)^2^		106.08 ± 12.80	101.71 ± 12.61	106.53 ± 12.31	0.286
Smoking during pregnancy^3^	No	85.71%	90.00%	91.43%	0.746
	Yes	14.29%	10.00%	8.57%	
Alcohol consumption during pregnancy^3^	No	95.24%	100%	94.29%	0.384
	Yes	4.76%	0.00%	5.71%	
Type of delivery^3^	Eutocia	67.92%	54.17%	73.08%	0.156
	Dystocia	15.09%	12.50%	0.00%	
	Cesarean	16.99%	33.33%	26.92%	
Neonate					
Gestational age (wks)^2^		39.58 ± 1.16	39.37 ± 1.43	39.87 ± 1.38	0.218
Birth weight (g)^2^		3329 ± 385	3269 ± 516	3492 ± 481	0.065
Birth length (cm)^2^		50.53 ± 1.48	50.00 ± 1.98	51.01 ± 2.41	0.119
Apgar 1′^4^		9(0)	9(0)	9(0)	0.262
Apgar 5′^4^		10(0)	10(0)	10(0)	0.978
Sex^3^	Boy	49.30%	48.89%	60.00%	0.495
	Girl	50.70%	51.11%	40.00%	
Type of feeding at 3 months^3^	Breast-feeding	61.19%	58.97%	37.50%	0.060
	Mixed	16.42%	17.95%	15.00%	
	Infant formula	22.39%	23.08%	47.50%	

*GWG* Gestational Weight Gain, *GDM* Gestational Diabetes Mellitus, *P* Level of significance for overall differences between PREOBE-groups. ^1^Mean ± Standard Deviation for normally distributed continues variables; ^2^Percentage for categorical variables; ^3^Median and Interquartile Range for non-normally distributed continuous variables

Statistical analysis performed: ANOVA: Analysis of variance for normally distributed variables. Kruskal–Wallis rank-sum test for non-normal continuous variables. Chi-square test for categorical variables. Values who do not share the same suffix (abc) are significantly different in a Bonferroni post hoc test. Bold: *p*-value < 0.05

### Effects of maternal prepregnancy BMI on children’s CBCL scores at 3.5 years old

The effects of maternal prepregnancy overweight or obesity on children’s CBCL scores at 3.5 years of age are shown in Table 2. We found that children born to overweight mothers showed higher scores in anxiety (p = 0.027) and total problems (p = 0.039) than children born to normoweight mothers, although significance was lost after adjustment for confounders (weight gain during pregnancy, maternal educational level and maternal IQ). Moreover, differences between children’s scores were seen in anxiety/depression (p = 0.044) and internalizing problems (p = 0.048); however, the p values did not remain significant after a Bonferroni post hoc test (p = 0.083 and p = 0.199, respectively). Additionally, the results for emotionally reactive and externalizing problem scores were not significant (p = 0.056 and p = 0.062, respectively), even after adjustment for confounding variables (p = 0.065 and p = 0.199, respectively).

**Table 2:** Effects of maternal pre-pregnancy BMI on children’s CBCL scores at 3.5 years of age

CCL Scores at 3.5 years	Normal weight (n = 71)	Overweight (n = 45)	Obese (n = 40)	P_unadj_	P_adj_
Emotionally reactive	54.80 ± 5.85	57.51 ± 6.74	56.83 ± 6.40	0.056	0.065
Anxious/depressed	54.68 ± 5.54^a^	57.47 ± 7.36^a^	56.65 ± 6.18^a^	**0.044**	0.083
Somatic complaints	55.37 ± 6.45	57.56 ± 7.72	56.68 ± 6.91	0.242	0.640
Withdrawn	56.48 ± 6.52	58.98 ± 7.88	56.13 ± 6.31	0.098	0.532
Sleep problems	55.92 ± 8.10	57.33 ± 8.06	55.75 ± 6.15	0.547	0.296
Attention problems	53.66 ± 5.05	54.96 ± 4.71	54.20 ± 4.60	0.376	0.725
Aggressive behaviour	53.46 ± 3.96	55.31 ± 5.90	55.30 ± 5.25	0.137	0.234
Internalizing problems	52.99 ± 9.00^a^	57.27 ± 10.16^a^	55.10 ± 9.40^a^	**0.048**	0.199
Externalizing problems	50.25 ± 7.53	53.29 ± 7.91	53.05 ± 7.60	0.062	0.345
Total problems	52.04 ± 8.11^a^	56.04 ± 9.17^b^	54.90 ± 8.85^a,b^	**0.039**	0.243
Affective problems	55.93 ± 6.11	58.13 ± 7.52	56.75 ± 6.09	0.213	0.387
Anxiety problems	54.83 ± 6.44^a^	58.33 ± 8.91^b^	57.88 ± 7.85^a,b^	**0.027**	0.472
Pervasive developmental problems	56.21 ± 6.44	58.78 ± 7.64	56.85 ± 7.13	0.153	0.913
Attention deficit/ hyperactivity problems	53.35 ± 4.73	54.98 ± 4.91	55.10 ± 5.50	0.114	0.758
Oppositional defiant problems	53.63 ± 4.22	54.78 ± 6.30	55.90 ± 5.80	0.241	0.378

Data are expressed as Mean ± Standard Deviation

Punadj = Level of significance unadjusted—Analysis of variance (ANOVA). Values not sharing the same suffix (ab) were significantly different in a Bonferroni post hoc test

Padj = Level of significance adjusted by potential confounders—Analysis of covariance (ANCOVA) for the group differences using univariate general linear model including main effects from the following possible confounder: Weight gain during pregnancy, maternal educational level and maternal IQ (n = 156)

Bold: *p*-value < 0.05

Subsequently, we categorized the CBCL children’s scores according to normal, borderline and clinical pathology outcomes (Table 3). In general, children born to overweight or obese mothers more frequently showed scores classified as borderline or clinical pathology. The analysis showed that children born to overweight mothers presented more borderline anxiety/depression (p = 0.018) and clinical pathology such as externalizing problems (p = 0.020) and total problems (p = 0.031) than children born to healthy normoweight mothers. Concerning oppositional defiant problems, children born to normoweight mothers were more frequently classified as normal than children born to overweight mothers (p = 0.008). Furthermore, children born to obese mothers presented more borderline anxiety problems (p = 0.006) than those born to healthy normoweight mothers.

**Table 3:** Effects of maternal pre-pregnancy overweight or obesity on children’s CBCL scores at 3.5 years of age compared to those born to healthy normoweight pregnant women (controls)

		**Normal weight (n = 71) (%)**	**Overweight (n = 45) (%)**	**Obese** **(n = 40) (%)**	**p**
Emotionally reactive	Normal	87.32	77.78	85.00	0.276
	Borderline	12.68	22.22	12.50	
	Clinical Pathology	0.00	0.00	2.50	
Anxious/depressed	Normal	92.96^a^	75.56^b^	90.00^a,b^	**0.018**
	Borderline	4.23^a^	22.22^b^	10.00^a,b^	
	Clinical Pathology	2.82	2.22	0.00	
Somatic complaints	Normal	88.73	77.78	77.50	0.210
	Borderline	8.45	13.33	20.00	
	Clinical Pathology	2.82	8.89	2.50	
Withdrawn	Normal	94.37^a^	80.00^a^	90.00^a^	**0.039**
	Borderline	0.00^a^	2.22^a^	5.00^a^	
	Clinical Pathology	5.63^a^	17.78^a^	5.00^a^	
Sleep problems	Normal	88.73	82.22	95.00	0.303
	Borderline	1.41	4.44	2.50	
	Clinical Pathology	9.86	13.33	2.50	
Attention problems	Normal	92.96	95.56	97.50	0.945
	Borderline	5.63	4.44	2.50	
	Clinical Pathology	1.41	0.00	0.00	
Aggressive behaviour	Normal	98.59	88.89	95.00	0.055
	Borderline	1.41	11.11	5.00	
	Clinical Pathology	0.00	0.00	0.00	
Internalizing problems	Normal	76.06	51.11	67.50	0.055
	Borderline	8.45	15.56	5.00	
	Clinical Pathology	15.49	33.33	27.50	
Externalizing problems	Normal	94.37^a^	77.78^b^	77.50^b^	**0.020**
	Borderline	2.82	6.67	12.50	
	Clinical Pathology	2.82^a^	15.56^b^	10.00^a,b^	
Total problems	Normal	84.51^a^	60.00^b^	65.00^a,b^	**0.031**
	Borderline	7.04	11.11	10.00	
	Clinical Pathology	8.45^a^	28.89^b^	25.00^a,b^	
Affective problems	Normal	90.14	84.44	92.50	0.378
	Borderline	5.63	2.22	2.50	
	Clinical Pathology	4.23	13.33	5.00	
Anxiety problems	Normal	92.96^a^	80.00^a,b^	75.00^b^	**0.006**
	Borderline	0.00^a^	0.00^a,b^	10.00^b^	
	Clinical Pathology	7.04	20.00	15.00	
Pervasive developmental problems	Normal	85.92	71.11	82.50	0.393
	Borderline	8.45	17.78	10.00	
	Clinical Pathology	5.63	11.11	7.50	
Attention deficit/ hyperactivity problems	Normal	95.77	95.56	90.00	0.366
	Borderline	2.82	2.22	10.00	
	Clinical Pathology	1.41	2.22	0.00	
Oppositional defiant problems	Normal	100.00^a^	88.89^b^	92.50^a,b^	**0.008**
	Borderline	0.00	6.67	7.50	
	Clinical Pathology	0.00	4.44	0.00	

Data are shown as percentages and p-values were obtained after Chi square test. Values who do not share the same suffix (ab) are significantly different in a Bonferroni post hoc test. Bold: *p*-value < 0.05

### Impact of GDM on children’s behavioral problems at 3.5 years old

Additionally, we studied the impact of the development of GDM in overweight, obese or normoweight pregnant women on children’s CBCL scores. The statistical analysis revealed significant differences only in offspring of the obese mothers group, as the children born to obese mothers with GDM presented higher scores in aggressive behavior (p = 0.008) and oppositional defiant problems (p = 0.004) than the children born to obese mothers without GDM (see Additional file 1: Table S1). Moreover, when we performed the analysis of CBCL scores by categorizing into clinical clusters, we found that the children born to obese mothers with GDM presented more clinical pathology regarding externalizing problems (p = 0.015) and more borderline ADHD symptoms (p = 0.011) than the children born to obese mothers without GDM (see Additional file 2: Table S2).

### Effects of maternal metabolic status and potential confounders on behavioral development in PREOBE children at 3.5 years of age

Logistic regression models, calculating the OR and 95% CI for CBCL scores of children born to obese and overweight groups, respectively, against the normoweight group as a reference, are presented in Additional file 3: Table S3. We found an increased risk in children born to overweight and obese mothers vs. those children born to normoweight mothers of having externalizing problems (OR 4.786, 95% CI 1.400–16.364, p = 0.013; OR 4.863, 95% CI 1.390–17.014, p = 0.013, respectively), total problems (OR 3.636, 95% CI 1.513–8.740, p = 0.004; OR 2.937, 95% CI 1.178–7.326, p = 0.021, respectively) and anxiety problems (OR 3.300, 95% CI 1.028–10.592, p = 0.045; OR 4.400, 95% CI 1.383–13.994, p = 0.012, respectively). A similar increased risk was seen in children born to overweight vs. normoweight mothers regarding the anxiety/depression score (OR 4.271, 95% CI 1.372–13.289, p = 0.012), withdrawn behavior score (OR 4.187, 95% CI 1.205–14.550, p = 0.024) and internalizing problems score (OR 3.038, 95% CI 1.366–6.757, p = 0.006). Nevertheless, when the logistic regression models were adjusted by confounding factors (weight gain during pregnancy, maternal educational level and maternal IQ), these differences disappeared.

### Correlation analysis between gut microbiota composition in children at 18 months of life and their CBCL scores at 3.5 years

**Fig. 2:**
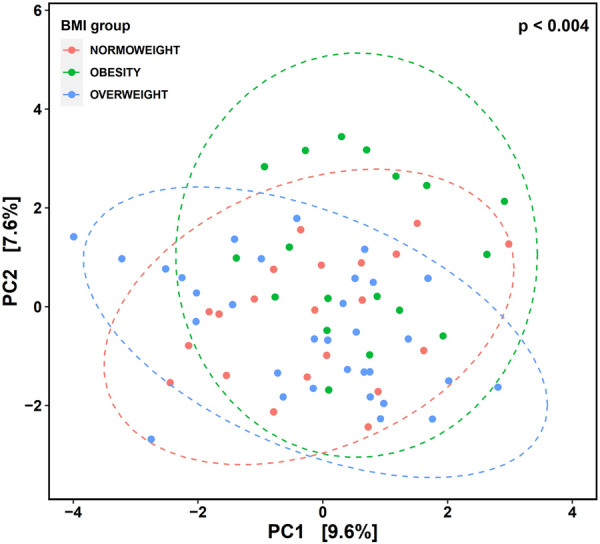
Principal component analysis based on Aitchison distance of microbial community composition in 18-moths old infants grouped by mother pre-gestational BMI: normoweight, overweight and obese. Each colored point represents a sample. Distance between samples on the plot represents differences in microbial community composition at genus level, with 17.2% of total variance being explained by the first two components

We initially examined gut microbiota covariation with CBCL scales in the context of known microbiota covariates, which included maternal age, smoking during pregnancy (yes/no), alcohol consumption during pregnancy (yes/no), neonate weight, gender, type of delivery (C-section or vaginal), gestational diabetes (yes/no), maternal pregestational BMI groups (normoweight, overweight and obese) and type of feeding up to the third month. We determined the proportion of interindividual variation in microbiota composition explained by covariates using a dbRDA at the genus level with Aitchison distance. This confounder analysis revealed a significant association with maternal pregestational BMI, which was considered in downstream statistical analyses to avoid covariate effects (Fig. 2).

**Fig. 3:**
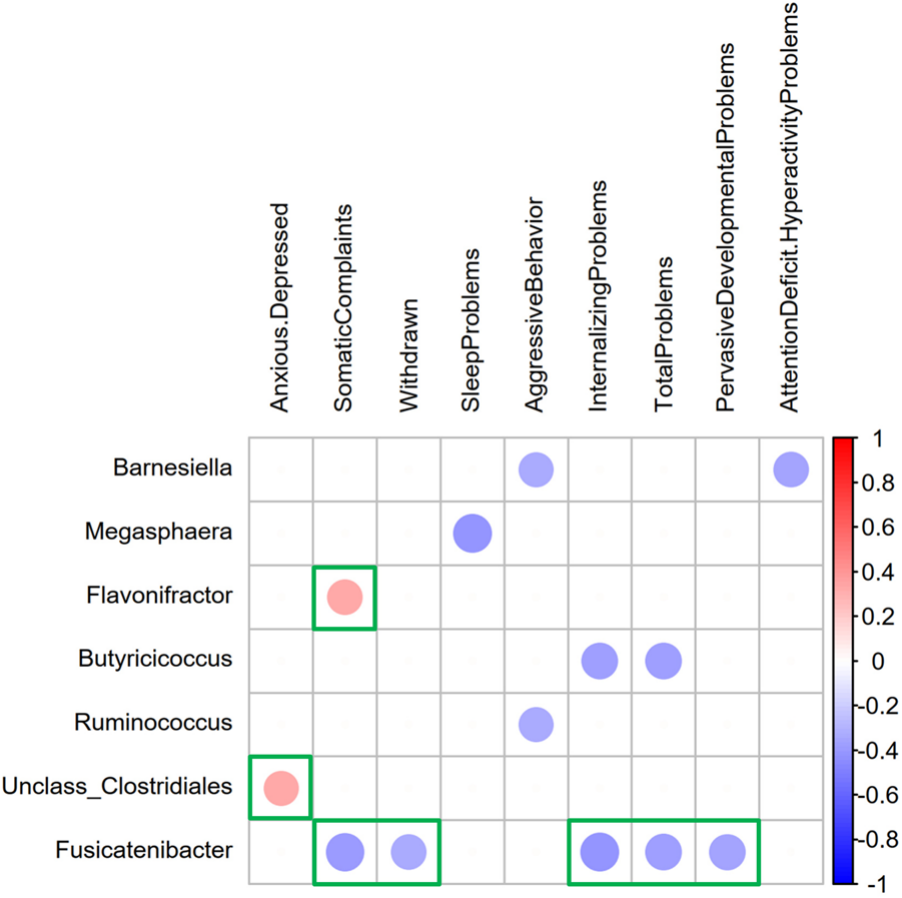
Heatmap diagram of significant correlations between gut microbial composition and CBCL scale. Green squares represent significant associations by fitting a generalized linear model (GLM) between taxa and CBCL scores after partially out the effect of microbiota covariables

Next, we identified associations of infant gut microbiota at the genus level with CBCL scales at 3.5 years (Spearman’s correlation, Bonferroni FDR < 0.01, Fig. 3). The internalizing problems scores were negatively correlated with *Fusicatenibacter* and *Butyricicoccus* within Firmicutes. Within the subscales, the anxiety/depression and somatic complaints scores were positively correlated with an unclassified genus in *Clostridiales (unclass_Clostridiales*) and with *Flavonifractor*, respectively, both belonging to *Firmicutes*. Somatic complaints, emotionally reactive problems, and withdrawn behavior scores were negatively correlated with *Fusicatenibacter*. On the “externalizing problems scale”, the aggressive behavior scores were negatively correlated with *Barnesiella (Bacteroidetes)* and *Ruminococcus (Firmicutes)*. On independent scales, the sleep problems scores were negatively correlated with *Megasphaera (Firmicutes)*. Attention deficit hyperactivity problems and aggressive behavior scores were negatively correlated with *Barnesiella*. Pervasive developmental problems scores were negatively correlated with *Fusicatenibacter*. Interestingly, the overall total problem scores were negatively correlated with *Fusicatenibacter* and *Butyricicoccus*.

Fitting GLMs between CBL subscales and microbial genera (FDR < 0.05; Fig. 3) while screening the maternal pregestational BMI covariate contribution confirmed significant correlations between the abundances of three genera and CBCL scores. An inverse association with mental health problems remained largely significant for the *Fusicatenibacter* genus. Negative correlations between *Fusicatenibacter* abundances were confirmed with total problems, internalizing problems and its subscales somatic complaints and withdrawn behavior, and pervasive developmental problems. Positive correlations between the abundances of *unclass*_*Clostridiales* and *Flavonifractor* and anxiety/depression and somatic complaints subscales, respectively, remained significant.

### Influences of maternal pregestational BMI on breastfeeding and their consequences on behavioral development in PREOBE children at 3.5 years of age and gut microbiota composition at 18 months

**Fig. 4:**
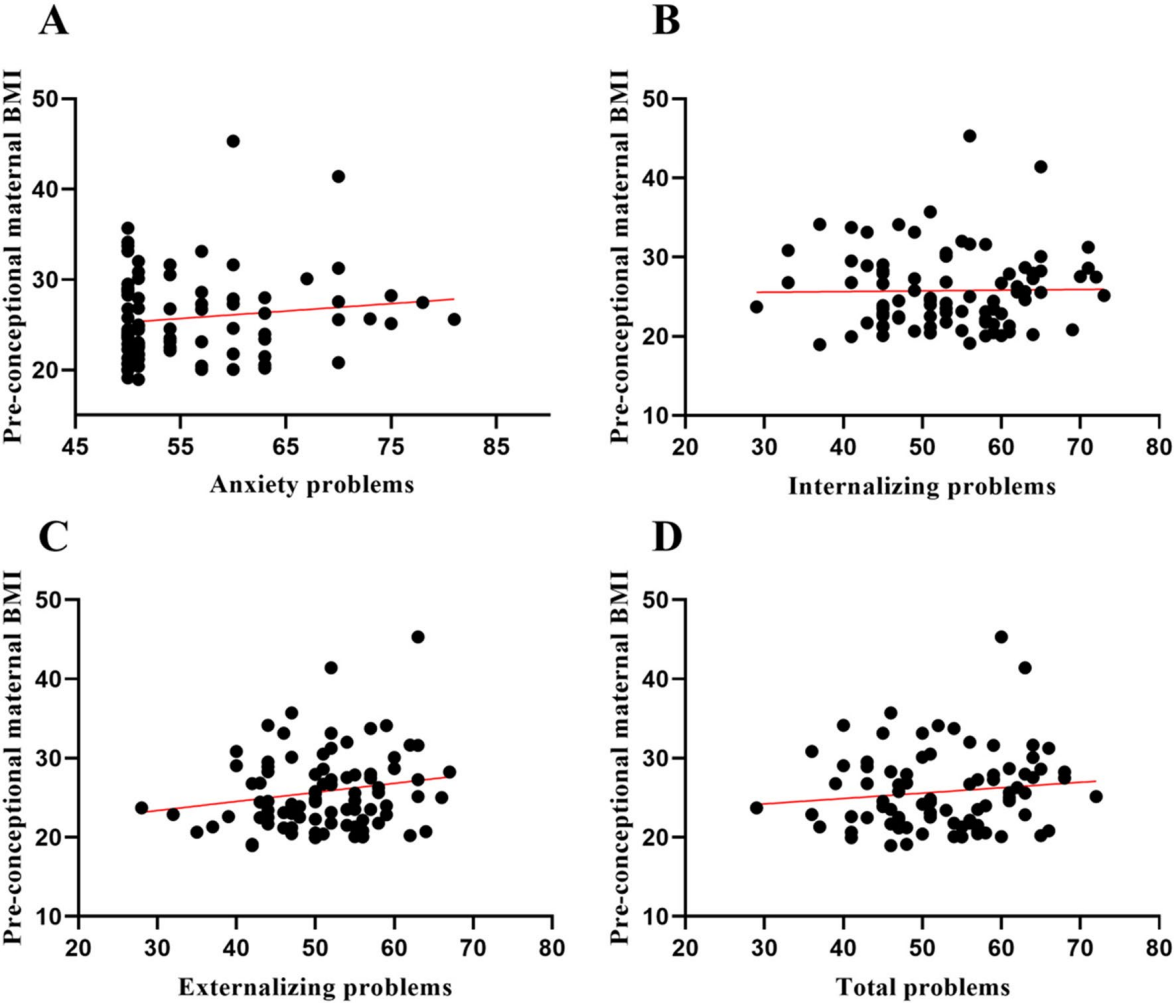
Spearman correlation analysis between pre-conceptional maternal BMI and anxiety (**A**), internalizing (**B**), externalizing (**C**) and total (**D**) problems. rs: correlation coefficient Rho Spearman

Finally, we tested whether maternal pregestational BMI had any effects on breastfeeding and its association with later behavioral development. Remarkably, Spearman’s correlation analysis revealed that higher maternal pregestational BMI was associated with higher anxiety (rs = 0.321; p = 0.003), internalizing (rs = 0.291; p = 0.006), externalizing (rs = 0.255; p = 0.018) and total problems (rs = 217; p = 0.045) in their children at 3.5 years old only in the group of children who were breastfed during their first 3 months of life. No correlations were found in the group of children fed with infant formula or mixed feeding (Fig. 4).

**Fig. 5:**
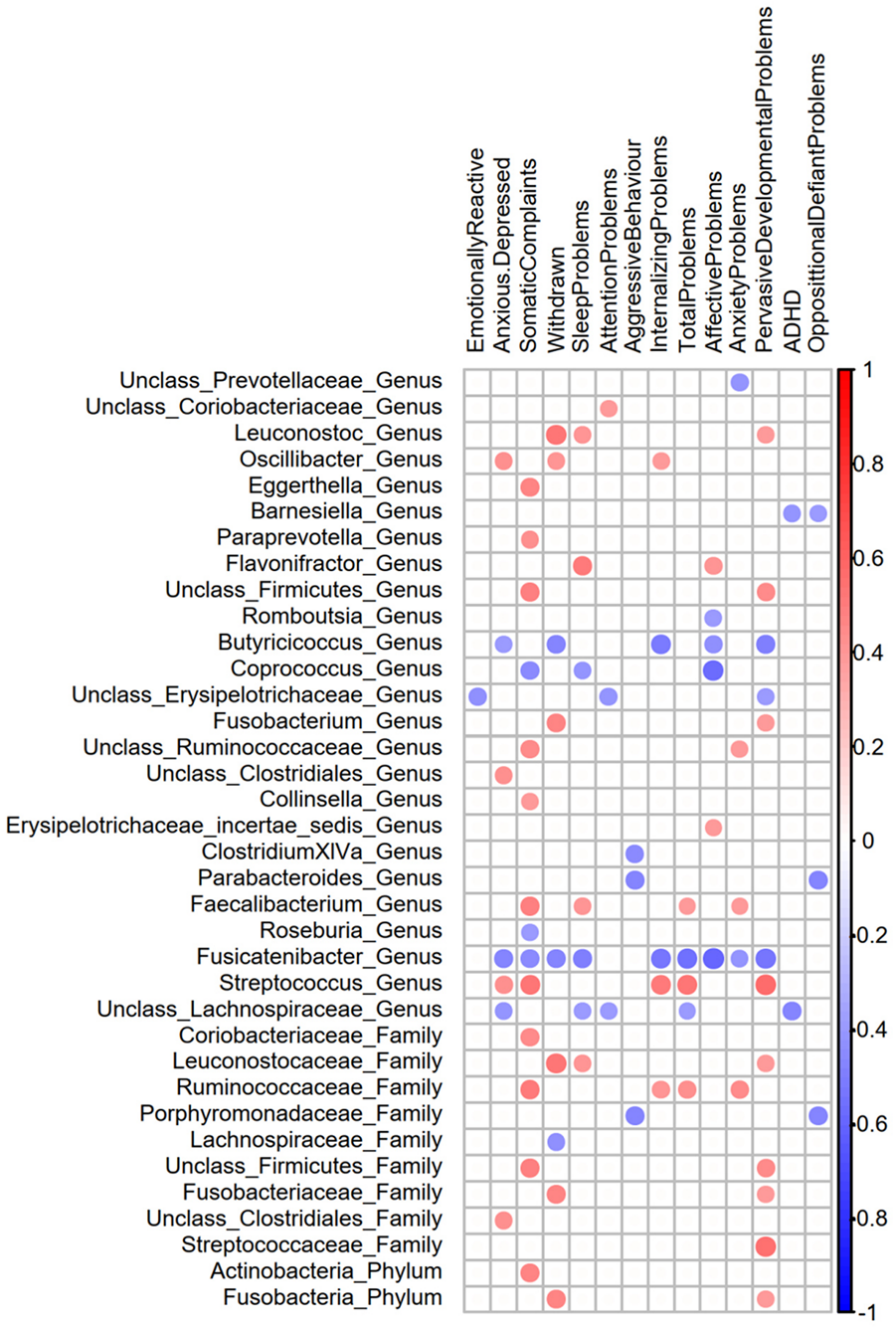
Heatmap diagram of significant correlations between gut microbial composition from children born to obese mothers with breastfeeding and CBCL scale

To identify the possible gut bacteria that could influence these results, we analyzed the gut microbiota composition of children at 18 months born to obese/overweight mothers who were exclusively breastfed during the first 3 months of life. At the phylum level, we detected a positive correlation between Actinobacteria and somatic complaints, *Fusobacteria* and withdrawn behavior and *Fusobacteria* and pervasive developmental problems. At the family level, 6 families (*Coriobacteriaceae*, *Leuconostocaceae*, *Ruminococcaceae*, *Unclass_Firmicutes*, *Fusobacteriaceae* and *Streptococcaceae*) were positively correlated with somatic complaints, withdrawn behavior, sleep problems, internalizing problems, pervasive developmental problems, total problems and anxiety/depression. Finally, we detected positive and negative correlations between all CBCL subscales and several genera, highlighting a positive correlation between *Flavonifractor* and sleep problems and negative correlations between *Fusicatenibacter* and somatic complaints, affective problems and pervasive developmental problems (Fig. 5).

## Discussion

Obesity before and during pregnancy is the most important cause of increased systemic inflammation in the fetal compartment, and exposure to a proinflammatory milieu might produce alterations in brain structure and later neurocognitive function and psychopathology [66–68]. Experimental studies in animals concluded that exposure to high levels of proinflammatory cytokines produced negative consequences in neurogenesis, apoptosis, neurotransmitter levels and myelination [69], leading to a decrease in gray and white matter volumes, abnormal development of fetal brain and later behavioral problems [70], and even increased risk for neuropsychiatric disorders [71]. In this regard, some studies in animals showed that the offspring of mothers who are obese during pregnancy showed reduced central serotonergic and dopaminergic signaling in brain regions (nucleus accumbeus, hippocampus and prefrontal cortex) associated with cognitive development and psychiatric problems in humans [72–74].

In the present study, we provide evidence that children born to obese and overweight mothers presented higher scores in the CBCL test and increased risk for developmental behavior problems at 3.5 years old than those born to healthy normoweight mothers. Recently, a meta-analysis supported the idea that offspring born to mothers who were overweight or obese prior to pregnancy have an increased risk of compromised neurodevelopmental outcomes, such as ADHD, autism spectrum disorder developmental delay and emotional/behavioral problems [39]. Moreover, Jo. et al. determined that children whose mothers were severely obese before pregnancy had an increased risk of unfavorable development, such as emotional symptoms, peer problems, ADHD, autism or developmental delay [75]. Another study showed that learning and behavioral disabilities, as well as ADHD, autism, pervasive development disorder, oppositional defiant disorder or another developmental delay, occurred more often in children who were born to severely obese mothers [76]. In addition, Mina et al*.* showed that 3- to 5-year-old children born to very severely obese mothers have higher scores for externalizing and total problems, anxiety/depression, aggressive behavior and other syndromes and more DSM-oriented affective problems, anxiety and ADHD problems in CBCL [77].

Affective problems, including major depressive disorder and dysthymic disorder, are the most common mental problems in children and adolescents [78, 79]. Recent studies have shown that factors present in fetal life could affect the development of common mental disorders, such as depression [80, 81]. Empirical data suggest that obesity and metabolic disorders, such as GDM, are associated with a higher risk of depression. Recent studies suggest a possible link between maternal metabolic conditions and programming of the fetal brain in the uterus, which could predispose the fetus to suffering from emotional problems in childhood [82]. Robinson et al*.* confirmed the relationship between maternal prepregnancy BMI (obesity and overweight) and the development of affective and emotional problems [78]. Additionally, maternal prepregnancy obesity and overweight were found to predict a higher risk for inattention and emotional regulation problems when children were 5 years old [83].

Moreover, interestingly, our results indicate that children born to obese mothers with GDM presented higher scores in psycho-behavior problems than children born to obese mothers without GDM or overweight/healthy normoweight mothers who developed GDM. Therefore, maternal *diabesity* represents the worst-case scenario for the fetus, increasing the odds of developing behavioral problems during childhood. Our results seem to be in agreement with other previously published studies showing that lower IQ, language disability, attention problems, impulsivity, and behavioral problems are linked to GDM [84, 85]. In addition, other studies found that children of diabetic mothers had higher rates of ADHD symptoms [42, 86]. The intrauterine conditions associated with childhood obesity are characterized by a series of prenatal factors, such as suboptimal maternal diet and/or nutritional deficiencies, diabetes mellitus, psychosocial stress, increased levels of proinflammatory cytokines, and obstetric complications. These factors are associated with deficits in brain growth in the offspring and are attributed to fetal programming, including brain inflammation, behavioral alterations in the offspring and later mental health [31, 87, 88]. The maturing brain is a target for these environmental insults, and anything affecting the developing brain, such as elevated circulating levels of glucose or fatty acids, has the potential to determine childhood and adult behavior [89, 90]. Particularly, in obese and diabetic pregnant women, an increased inflammatory milieu appears during gestation that might represent a biological condition involved in the genesis and development of behavioral and psychological problems in the offspring. Among other possible mechanisms proposed to underlie the risk of neurodevelopmental morbidity are the dysregulation of leptin signaling in the developing brain, serotonergic and dopaminergic signaling and impaired reward circuitry or alteration of brain-derived neurotrophic factor-mediated synaptic plasticity [43].

Psycho-behavior research has provided evidence of an association between maternal prepregnancy BMI and emotional and behavioral problems in children [91], and a higher risk of externalizing (ADHD and aggressive behavior) and internalizing problems are related to prepregnancy adiposity [92]. However, as the higher risk for externalizing problems appears early during development, some recent research has shown the increasing risk for internalizing problems later in life. It is not yet clear whether the delayed expression of internalizing symptoms is caused by stress linked to externalizing problems in early life, programming of internalizing problems during pregnancy or heterotypic continuity [87]. Nevertheless, our data showed higher scores in internalizing symptoms such as anxiety/depression and externalizing problems in children 3.5 years old born to overweight and obese mothers with GDM, respectively, suggesting that psycho-emotional and behavioral problems may be programmed in early life. It is important to note that maternal psychosocial variables, such as maternal IQ, educational level, and breastfeeding, were associated with lower psycho-behavioral problems, influencing child behavior development more frequently.

There is increasing evidence on the influence of the type of feeding during the first months of life on infant development as well as on later health in adult life[7]. During the first 1000 days of life, the intensity of growth makes the brain particularly vulnerable to adverse nutritional stimuli and has a direct impact on cognitive and behavioral development [93], and this period is a critical window in the establishment of the gut microbiota community, which has been linked with later neurodevelopment skills and obesity status [94]. Breastfeeding is the gold standard of nutrition for optimal development, and the benefits on cognitive function are clear [95], but there are fewer studies about its role in emotional regulation and behavior development. In this regard, breastfeeding has been associated with a lower risk of behavioral problems in childhood. Parker et al. found that breastfeeding absence was associated with increased internalizing, externalizing, and overall behavioral problems as well as the diagnosis of ADHD [96]. In another study, children who were breastfed and whose mothers actively engaged with them showed the lowest risk of internalizing problems at 6 years old [97]. However, some studies have not found an association between breastfeeding and behavioral problems during early childhood [98]. Kwok et al*.* found inconsistent associations between breastfeeding and several early adolescent mental health factors, where confounding factors, such as socioeconomic status and maternal educational level, play an important role in the establishment of a good behavioral system and mental health [99]. Along these lines, several studies have shown that a large number of social and parental educational factors might influence child development independently of the breastfeeding effect [95]. Wigg et al. noted that when confounders such as social advantage, maternal education and intelligence, and the quality of a child’s developmental experiences were taken into account, the differences between bottle-fed and breastfed groups disappeared [100].

Most likely, there is an association between social, genetic and nutritional factors that could be essential for optimal brain development. The benefits of breastfeeding could be related to a stronger psychological attachment between mother and child neurodevelopment and not only to breast milk intake. Quinn et al. found that children who were breastfed presented better scores in language than formula-fed children, but this association was reduced though remaining significant when a large number of confounding social and parental factors were considered [95]. In this sense, milk from obese or gestational diabetic mothers might contain a lower abundance of protective factors than milk from normoweight mothers [101, 102]. Likewise, the omega-6/omega-3 ratio is increased, while the fatty acid (docosahexaenoic acid, eicosapentaenoic acid and docosapentaenoic acid) concentration and carotenoid (lutein) concentration are decreased [103]. Interestingly, and according to our results, breastfeeding in the offspring of overweight and obese mothers appears to be related to higher anxiety, internalizing problems, externalizing problems and total problems in children at 3.5 years old.

During early childhood, many behavioral problems that determine the development of mental pathologies in adult life, as well as higher rates of school dropout, substance abuse, problems with justice and suicide, can be identified. The emotional and psychological state of the child might be inferred through internalization problems, such as anxiety, depression, somatic complaints, withdrawn behavior or affective problems [104]. In contrast, externalizing problems manifest as aggressive behavior, opposite defiance or inattention. Thus, there is a clear distinction between externalizing and internalizing disorders. Despite the overlap between both, as children with internalizing problems might have disruptive behaviors with the environment and vice versa, children with externalizing problems could suffer several internal emotional problems. Therefore, it is necessary to perform a behavioral global study to understand its etiology and long-term consequences [105–107].

In addition, advances in 16S rDNA gene sequencing have recently shown the association of the gut microbiota with the pathophysiology of neurological disorders, such as anorexia nervosa, major depressive disorder (MDD), bipolar disorder, anxiety, psychosis, and schizophrenia. Fecal microbiota transplants from patients with psychiatric conditions resulted in the development of behavioral and physiological responses in germ-free mice, suggesting that the gut microbiota may be involved in neurological disorders and may serve as a biomarker[108]. Interestingly, evidence from epidemiological studies indicates that maternal prepregnancy obesity is also associated with increased risks for autism spectrum disorder, cognitive dysfunction, attention-deficit hyperactivity disorder, and other mental disorders [109]. In fact, our previous studies showed that maternal prepregnancy obesity may imprint a selective gut microbial composition during late infancy with distinct functional performances [47], and perhaps this factor would modulate the development of fine motor skills [110]. In the present study, we observed negative correlations between *Fusicatenibacter* and several CBCL scores. The *Fusicatenibacter* genus comprises one single cultured *Fusicatenibacter* species, *F. saccharivorans*, a strict anaerobic sugar fermenter and producer of propionate and acetate[111]. Dong et al*.* showed that the abundance of *Fusicatenibacter* was significantly lower in patients with MDD and general anxiety disorder, where a significant negative correlation between *Fusicatenibacter* abundance and thyroid hormone FT4 levels was observed[112]. Valles-Colomer et al*.* identified significant associations between the abundances of 10 genera and quality-of-life scores, including both mental and physical scores, where *Fusicatenibacter* was positively correlated with quality-of-life scores. Nevertheless, Medawar et al. observed a positive correlation between *Fusicatenibacter* abundances and unhealthy eating behavior, higher subjective hunger ratings and lower fecal concentrations of propionate and acetate [113]. Another genus linked to neurodevelopmental disorders was *Flavonifractor,* whose abundances were positively correlated with somatic complaints in our study. While Rothenberg et al*.* observed a positive correlation between *Flavonifractor* abundance and mental developmental index scores in children at 3 years of age [114], Luna et al*.* conducted a study showing higher levels of *Clostridiales*, including *Flavonifractor plautii*, in children with autism spectrum and functional gastrointestinal disorders[115]. Likewise, in our study, an unassigned genus within *Clostridiales* was positively correlated with anxiety/depression scores. Bacteria within *Clostridiales* are predominant members of the gut microbiota that are more abundant in children with neurodevelopmental disorders[116]. Furthermore, Rhee et al*.* analyzed the association of serum microbial DNA composition with depressive and anxiety symptoms in patients, observing a positive association between the *Desulfovibrionaceae* family and *Clostridiales Family XIII* with the total Beck Anxiety Inventory score[117]. Nevertheless, in adults, Li et al*.* showed a significant depletion of 6 genera within *Clostridiales* in multiple psychiatric diseases that was associated with dysfunction in amino acid and carbohydrate metabolism [118]. Our results suggest an association between gut microbiota at 18 months of life and CBCL scores in 3.5-year-old children that varies by population characteristics, type of disorder and timing of microbiota assessment. Further accurate and reliable evidence is needed to clarify the potential role of early life gut microbiota imprinting and maturation in children with neurodevelopmental disorders.

On the other hand, to improve prevention and intervention strategies, early detection of psychological and social factors that contribute to the development and maintenance of overweight and obesity, especially during pregnancy, is necessary because of the long-term consequences on children’s health. In this regard, our results highlight the importance of studying the influence of prepregnancy obesity and GDM on children’s future psycho-behavior and central nervous system development, considering their gut microbiota composition as a key modulator.

In summary, our current study helps to fill the gap in examining the relationship between prepregnancy weight status and child behavior development and confirms previous results shown in other studies, as there is not enough evidence about the impact of maternal metabolic state on behavior problems in children between 2 and 5 years old, especially in GDM mothers. Moreover, our results provide evidence that children whose mothers are obese and present GDM have an increased risk of developing behavioral problems. Furthermore, we provide evidence that the early gut microbiota composition in infants is a possible behavioral modulator for the future design of preventive strategies. Finally, the aforementioned influences of nutritional and maternal sociodemographic factors might help to clarify the etiology of behavioral problems during childhood. In this regard, it is important to note that human milk of overweight and obese mothers seems to be related to higher behavioral problems in children at 3.5 years old.

### Strengths and limitations

The main strength of this study is its longitudinal design, which allowed long-term monitoring. An important issue has been the possibility of performing gut microbiota analysis at 18 months and behavioral assessment in this special cohort, which allowed us to develop these pilot studies to formulate new hypotheses. Furthermore, the presence of a specific group of mothers who developed GDM made it possible to study the effects of GDM development according to preconceptional BMI. The use of CBCL scales, a highly reliable and valid measure of childhood behavior, permitted us to arrive at important conclusions. All of the children evaluated were healthy, and maternal IQ and education level were used as confounding factors. Notably, the present study underlined the importance of optimal implantation of the intestinal microbiota during the first months of life in the development of behavioral alterations in children, which can be associated with different mental illnesses later in life. In addition, this study considers the intestinal microbiota as a potential biomarker of long-term problems related to mental health.

Among the limitations of the study, we should note that we have not included data about the mother's mental health status during pregnancy or stress, anxiety or depression, and other important variables such as diet or nutritional deficiencies. Moreover, we had no data on socioeconomic status at the moment of the evaluation, although other studies accounted for it and found it to be a significant predictor of mental health problems in children [119]. In addition, although our results regarding the offspring of obese mothers with GDM are in agreement with previously published studies [42, 86], caution should be taken because of the relatively small sample size of our groups.

## Conclusions

According to our results, maternal overweight and obesity during pregnancy are significantly associated with elevated levels of behavior problems. Being obese with GDM during pregnancy increases behavioral problems in the offspring. This effect is not observed in overweight or normoweight mothers. Furthermore, the type of feeding during the first months of life, early gut microbiota composition and maternal psychosocial variables more commonly influence child behavior development. The results link early life gut microbiota composition with later mental health in children and state the importance of maternal metabolic status, suggesting a fetal programming of mental health and different nutritional and environmental factors in the causation of behavioral problems in children. Future research is needed to verify and clarify the mechanisms behind the observed associations.

## Supplementary Information

**Additional file 1: Table S1.** Effects of development of gestational diabetes mellitus on children’s CBCL scores at 3.5 years old.

**Additional file 2: Table S2.** Effects of development of gestational diabetes mellitus on children’s CBCL clinical-clusters at 3.5 years old.

**Additional file 3: Table S3.** Logistic regression models assessing the odds of having the CBCL scores at 3.5 years old.

## Data Availability

The data presented in this study are available on request from the corresponding author. The data are not publicly available due to ethical reasons.

## Abbreviations

ADHD: Attention-deficit/hyperactivity disorder
ANCOVA: Univariate general linear model;
ANOVA: Analysis of variance
BMI: Body mass index
CBCL: Child Behavior Checklist
CIs: Confidence intervals
dbRDA: Distance-based redundancy analysis
DNA: Deoxyribonucleic acid
FDR: False discovery rate
GDM: Gestational diabetes mellitus
GLM: Generalized linear model
HMOs: Human milk oligosaccharides
IQ: Intelligence quotient
LGA: Large for gestational age
NW: Normoweight
OB: Obese
ORs: Odds ratios
OV: Overweight
PCRs: Polymerase chain reactions
PPH: Postpartum hemorrhage
rRNA: Ribonucleic acid ribosomal
SCFA: Short-chain fatty acid
T2DM: Type 2 diabetes mellitus
