# Supplementary material for: Maternal weight, gut microbiota, and the association with early childhood behavior: the PREOBE follow-up study
Source: Child Adolesc Psychiatry Ment Health. 2023 Mar 21;17:41. doi: 10.1186/s13034-023-00589-9 (PMC10031971; doi:10.1186/s13034-023-00589-9)
Supplement: Supplementary file 1 — Additional file 1: Table S1. Effects of development of gestational diabetes mellitus on children’s CBCL scores at 3.5 years old. [file 13034_2023_589_MOESM1_ESM.docx]

**Table S1.** Effects of development of gestational diabetes mellitus on children’s CBCL scores at 3.5 years old

|  | **Normal weight (n=71)** | | **p** | **Overweight (n=45)** | | **p** | **Obese (n=40)** | | **p** |
| --- | --- | --- | --- | --- | --- | --- | --- | --- | --- |
|  | **NO GDM (n=51)** | **GDM**  **(n=20)** |  | **NO GDM**  **(n=31)** | **GDM**  **(n=14)** |  | **NO GDM**  **(n=26)** | **GDM**  **(n=14)** |  |
| Emotionally Reactive | 54.29±5.58 | 56.10±6.45 | 0.275 | 56.84±6.24 | 59.00±7.78 | 0.284 | 56.08±6.87 | 58.21±5.38 | 0.304 |
| Anxious/ Depressed | 54.69±5.08 | 54.65±6.72 | 0.983 | 57.10±7.47 | 58.29±7.32 | 0.560 | 56.19±5.82 | 57.50±6.94 | 0.533 |
| Somatic Complaints | 55.80±6.91 | 54.25±5.08 | 0.398 | 56.90±7.83 | 59.00±7.57 | 0.350 | 55.62±6.57 | 58.64±7.34 | 0.191 |
| Withdrawn | 56.47±6.39 | 56.50±7.03 | 0.987 | 59.03±8.04 | 58.86±7.81 | 0.938 | 55.92±5.55 | 56.50±7.75 | 0.803 |
| Sleep Problems | 56.55±8.68 | 54.30±6.29 | 0.265 | 58.35±8.42 | 55.07±6.93 | 0.183 | 55.08±4.96 | 57.00±7.98 | 0.448 |
| Attention Problems | 54.22±5.56 | 52.25±3.11 | 0.127 | 54.97±4.51 | 54.93±5.31 | 0.980 | 54.08±4.45 | 54.43±5.03 | 0.827 |
| Aggressive Behaviour | 53.24±3.91 | 54.05±4.12 | 0.522 | 54.61±5.07 | 56.86±7.40 | 0.150 | 53.81±4.12 | 58.07±6.11 | **0.008** |
| Internalizing Problems | 53.22±8.44 | 52.40±10.51 | 0.745 | 56.65±10.18 | 58.64±10.35 | 0.515 | 54.12±9.53 | 56.93±9.21 | 0.373 |
| Externalizing Problems | 50.16±7.47 | 50.50±7.87 | 0.865 | 52.55±7.58 | 54.93±8.68 | 0.336 | 51.69±6.86 | 55.57±8.48 | 0.128 |
| Total Problems | 52.08±8.00 | 51.95±8.57 | 0.955 | 55.71±9.00 | 56.79±9.83 | 0.699 | 53.54±8.92 | 57.43±8.43 | 0.176 |
| Affective Problems | 55.71±6.06 | 56.50±6.36 | 0.645 | 59.19±8.26 | 55.79±5.03 | 0.107 | 56.00±5.09 | 58.14±7.63 | 0.324 |
| Anxiety Problems | 55.14±6.27 | 54.05±6.95 | 0.591 | 58.10±9.12 | 58.86±8.73 | 0.758 | 57.54±8.22 | 58.50±7.37 | 0.705 |
| Pervasive Developmental  Problems | 56.65±6.62 | 55.10±5.98 | 0.405 | 58.81±7.52 | 58.71±8.19 | 0.968 | 56.35±7.36 | 57.79±6.84 | 0.537 |
| Attention Deficit/Hyperactiviy Problems | 53.57±5.08 | 52.80±3.75 | 0.559 | 55.13±5.28 | 54.64±4.13 | 0.762 | 54.00±4.27 | 57.14±6.99 | 0.059 |
| Oppositional Defiant Problems | 53.51±4.05 | 53.95±4.72 | 0.749 | 54.29±5.41 | 55.86±8.06 | 0.351 | 54.15±4.59 | 59.14±6.56 | **0.004** |

Data are expressed as Mean ± Standard Deviation. P =Analysis Univariate of variance. GDM: Gestational diabetes mellitus. Bold: *p*-value < 0.05.
