# Supplementary material for: Maternal weight, gut microbiota, and the association with early childhood behavior: the PREOBE follow-up study
Source: Child Adolesc Psychiatry Ment Health. 2023 Mar 21;17:41. doi: 10.1186/s13034-023-00589-9 (PMC10031971; doi:10.1186/s13034-023-00589-9)
Supplement: Supplementary file 2 — Additional file 2: Table S2. Effects of development of gestational diabetes mellitus on children’s CBCL clinical-clusters at 3.5 years old. [file 13034_2023_589_MOESM2_ESM.docx]

**Table S2.** Effects of development of gestational diabetes mellitus on children’s CBCL clinical-clusters at 3.5 years old

|  |  | **Normal weight (n=71)** | | **p** | **Overweight (n=45)** | | **p** | **Obese (n=40)** | | **p** |
| --- | --- | --- | --- | --- | --- | --- | --- | --- | --- | --- |
|  |  | **NO GDM (n=50)** | **GDM**  **(n=20)** |  | **NO GDM**  **(n=31)** | **GDM**  **(n=14)** |  | **NO GDM**  **(n=26)** | **GDM**  **(n=14)** |  |
| Emotionally Reactive | Normal | 90.20% | 80.00% | 0.245 | 83.87% | 64.29% | 0.244 | 84.62% | 85.71% | 1.000 |
|  | Borderline | 9.80% | 20.00% |  | 16.13% | 35.71% |  | 11.54% | 14.29% |  |
|  | Clinical Pathology | 0.00% | 0.00% |  | 0.00% | 0.00% |  | 3.85% | 0.00% |  |
| Anxious/ Depressed | Normal | 94.12% | 90.00% | 0.775 | 77.42% | 71.43% | 0.802 | 88.46% | 92.86% | 1.000 |
|  | Borderline | 3.92% | 5.00% |  | 19.35% | 28.57% |  | 11.54% | 7.14% |  |
|  | Clinical Pathology | 1.96% | 5.00% |  | 3.23% | 0.00% |  | 0.00% | 0.00% |  |
| Somatic Complaints | Normal | 86.27% | 95.00% | 0.833 | 80.65% | 71.43% | 0.737 | 80.77% | 71.43% | 0.495 |
|  | Borderline | 9.80% | 5.00% |  | 12.90% | 14.29% |  | 19.23% | 21.43% |  |
|  | Clinical Pathology | 3.92% | 0.00% |  | 6.45% | 14.29% |  | 0.00% | 7.14% |  |
| Withdrawn | Normal | 96.08% | 90.00% | 0.314 | 77.42% | 85.71% | 1.000 | 92.31% | 85.71% | 0.227 |
|  | Borderline | 0.00% | 0.00% |  | 3.23% | 0.00% |  | 7.69% | 0.00% |  |
|  | Clinical Pathology | 3.92% | 10.00% |  | 19.35% | 14.29% |  | 0.00% | 14.29% |  |
| Sleep Problems | Normal | 86.27% | 95.00% | 0.766 | 77.42% | 92.86% | 0.554 | 100.00% | 85.71% | 0.117 |
|  | Borderline | 1.96% | 0.00% |  | 6.45% | 0.00% |  | 0.00% | 7.14% |  |
|  | Clinical Pathology | 11.76% | 5.00% |  | 16.13% | 7.14% |  | 0.00% | 7.14% |  |
| Attention Problems | Normal | 90.20% | 100% | 0.520 | 96.77% | 92.86% | 0.530 | 100.00% | 92.86% | 0.350 |
|  | Borderline | 7.84% | 0.00% |  | 3.23% | 7.14% |  | 0.00% | 7.14% |  |
|  | Clinical Pathology | 1.96% | 0.00% |  | 0.00% | 0.00% |  | 0.00% | 0.00% |  |
| Aggressive Behaviour | Normal | 98.04% | 100% | 1.000 | 93.55% | 78.57% | 0.166 | 100.00% | 85.71% | 0.117 |
|  | Borderline | 1.96% | 0.00% |  | 6.45% | 21.43% |  | 0.00% | 14.29% |  |
|  | Clinical Pathology | 0.00% | 0.00% |  | 0.00% | 0.00% |  | 0.00% | 0.00% |  |
| Internalizing Problems | Normal | 76.47% | 75.00% | 0.730 | 54.84% | 42.86% | 0.757 | 69.23% | 64.29% | 1.000 |
|  | Borderline | 9.80% | 5.00% |  | 16.13% | 14.29% |  | 3.85% | 7.14% |  |
|  | Clinical Pathology | 13.73% | 20.00% |  | 29.03% | 42.86% |  | 26.92% | 28.57% |  |
| Externalizing Problems | Normal | 92.16% | 100% | 1.000 | 83.87% | 64.29% | 0.242 | 84.62% | 64.29% | **0.015** |
|  | Borderline | 3.92% | 0.00% |  | 3.23% | 14.29% |  | 15.38% | 7.14% |  |
|  | Clinical Pathology | 3.92% | 0.00% |  | 12.90% | 21.43% |  | 0.00%^b^ | 28.57%^a^ |  |
| Total Problems | Normal | 84.31% | 85.00% | 1.000 | 61.29% | 57.14% | 0.803 | 69.23% | 57.14% | 0.687 |
|  | Borderline | 7.84% | 5.00% |  | 12.90% | 7.14% |  | 7.69% | 14.29% |  |
|  | Clinical Pathology | 7.84% | 10.00% |  | 25.81% | 35.71% |  | 23.08% | 28.57% |  |
| Affective Problems | Normal | 90.20% | 90.00% | 0.167 | 77.42% | 100.00% | 0.212 | 96.15% | 85.71% | 0.118 |
|  | Borderline | 7.84% | 0.00% |  | 3.23% | 0.00% |  | 3.85% | 0.00% |  |
|  | Clinical Pathology | 1.96% | 10.00% |  | 19.35% | 0.00% |  | 0.00% | 14.29% |  |
| Anxiety Problems | Normal | 94.12% | 90.00% | 0.616 | 80.65% | 78.57% | 1.000 | 76.92% | 71.43% | 0.212 |
|  | Borderline | 0.00% | 0.00% |  | 0.00% | 0.00% |  | 3.85% | 21.43% |  |
|  | Clinical Pathology | 5.88% | 10.00% |  | 19.35% | 21.43% |  | 19.23% | 7.14% |  |
| Pervasive Developmental  Problems | Normal | 86.27% | 85.00% | 0.255 | 74.19% | 64.29% | 0.523 | 88.46% | 71.43% | 0.225 |
|  | Borderline | 5.88% | 15.00% |  | 12.90% | 28.57% |  | 3.85% | 21.43% |  |
|  | Clinical Pathology | 7.84% | 0.00% |  | 12.90% | 7.14% |  | 7.69% | 7.14% |  |
| Attention Deficit/ Hyperactivity  Problems | Normal | 94.12% | 100% | 1.000 | 93.55% | 100.00% | 1.000 | 100.00%^b^ | 71.43%^a^ | **0.011** |
|  | Borderline | 3.92% | 0.00% |  | 3.23% | 0.00% |  | 0.00%^b^ | 28.57%^a^ |  |
|  | Clinical Pathology | 1.96% | 0.00% |  | 3.23% | 0.00% |  | 0.00% | 0.00% |  |
| Oppositional Defiant Problems | Normal | 100% | 100% | - | 93.55% | 78.57% | 0.201 | 96.15% | 85.71% | 0.276 |
|  | Borderline | 0.00% | 0.00% |  | 3.23% | 14.29% |  | 3.85% | 14.29% |  |
|  | Clinical Pathology | 0.00% | 0.00% |  | 3.23% | 7.14% |  | 0.00% | 0.00% |  |

Data are percentage and p-values are Chi square test. Values who do not share the same sufﬁx (ab) are signiﬁcantly different in a Bonferroni post hoc test.

GDM: Gestational diabetes mellitus. Bold: *p*-value < 0.05.
