# Supplementary material for: Maternal weight, gut microbiota, and the association with early childhood behavior: the PREOBE follow-up study
Source: Child Adolesc Psychiatry Ment Health. 2023 Mar 21;17:41. doi: 10.1186/s13034-023-00589-9 (PMC10031971; doi:10.1186/s13034-023-00589-9)
Supplement: Supplementary file 3 — Additional file 3: Table S3. Logistic regression models assessing the odds of having the CBCL scores at 3.5 years old. [file 13034_2023_589_MOESM3_ESM.docx]

**Table S3.** Logistic regression models assessing the odds of having the CBCL scores at 3.5 years old

|  | **Overweight** | | | | **Obesity** | | | |
| --- | --- | --- | --- | --- | --- | --- | --- | --- |
|  | *Unadjusted* | | *Adjusted ^1^* | | *Unadjusted* | | *Adjusted ^1^* | |
|  | **OR (95% CI)** | **p** | **OR (95% CI)** | **p** | **OR (95% CI)** | **p** | **OR (95% CI)** | **p** |
| **CBCL SCORES AT 3.5 YEARS** |  |  |  |  |  |  |  |  |
| Emotionally Reactive | 1.968(0.730-5.304) | 0.181 | 1.594(0.472-5.384) | 0.453 | 1.216(0.399-3.705) | 0.731 |  | 0.918 |
| Anxious/ Depressed | 4.271(1.372-13.289) | **0.012*** | 3.757(0.987-14.295) | 0.052 | 1.467(0.370-5.807) | 0.585 | 1.299(0.243-6.949) | 0.760 |
| Somatic Complaints | 2.250(0.813-6.223) | 0.118 | 1.629(0.467-5.680) | 0.444 | 2.286(0.804-6.500) | 0.121 | 1.123(0.261-4.823) | 0.876 |
| Withdrawn | 4.187(1.205-14.550) | **0.024*** | 4.151(0.926-18.613) | 0.063 | 1.861(0.439-7.886) | 0.399 | 1.074(0.151-7.622) | 0.943 |
| Sleep Problems | 1.703(0.589-4.918) | 0.325 | 2.626(0.813-8.482) | 0.107 | 0.414(0.084-2.055) | 0.281 | 0.343(0.036-3.236) | 0.350 |
| Attention Problems | 0.614(0.114-3.308) | 0.570 | 0.206(0.018-2.400) | 0.207 | 0.338(0.038-3.004) | 0.331 | ---- | 0.998 |
| Aggressive Behaviour | 8.750(0.987-77.552) | 0.051 | ---- | 0.997 | 3.684(0.323-41.963) | 0.293 | ---- | 0.997 |
| Internalizing Problems | 3.038(1.366-6.757) | **0.006*** | 2.013(0.785-5.160) | 0.145 | 1.529(0.649-3.604) | 0.331 | 0.966(0.317-2.943) | 0.951 |
| Externalizing Problems | 4.786(1.400-16.364) | **0.013*** | 3.103(0.678-14.205) | 0.145 | 4.863(1.390-17.014) | **0.013*** | 3.699(0.760-17.997) | 0.105 |
| Total Problems | 3.636(1.513-8.740) | **0.004*** | 1.652(0.597-4.573) | 0.333 | 2.937(1.178-7.326) | **0.021*** | 2.062(0.688-6.181) | 0.196 |
| Affective Problems | 1.684(0.548-5.172) | 0.362 | 1.005(0.269-3.752) | 0.994 | 0.741(0.181-3.042) | 0.678 | 0.505(0.088-2.912) | 0.445 |
| Anxiety Problems | 3.300(1.028-10.592) | **0.045*** | 2.165(0.593-7.900) | 0.242 | 4.400(1.383-13.994) | **0.012*** | 2.814(0.724-10.939) | 0.135 |
| Pervasive Developmental Problems | 2.478(0.979-6.273) | 0.055 | 1.587(0.512-4.917) | 0.424 | 1.294(0.451-3.715) | 0.632 | 1.459(0.412-5.170) | 0.558 |
| Attention Deficit/ Hyperactivity Problems | 1.054(0.169-6.569) | 0.955 | 0.267(0.022-3.237) | 0.299 | 2.519(0.534-11.872) | 0.243 | 0.683(0.092-5.080) | 0.710 |
| Oppositional Defiant Problems | ---- | 0.997 | ---- | 0.997 | ---- | 0.997 | ---- | 0.997 |

ORs are presented for infants born to mothers with overweight or obesity respectively using infants born to mothers with normal weight as reference.

^1^ Logistic regression model adjusted including main effects from the following possible confounder: Maternal educational level, maternal IQ and weight gain during pregnancy.

^2^ ORs for a value of CBCL score.

Bold: *p*-value < 0.05.
